# Efficacy of Oncolytic Virus VV-GMCSF-Lact Against Immunocompetent Glioma

**DOI:** 10.3390/cells14201619

**Published:** 2025-10-17

**Authors:** Alisa Ageenko, Natalia Vasileva, Gaukhar Yusubalieva, Aleksandra Sen’kova, Alexander Romashchenko, Ilya Gubskiy, Fedor Zabozlaev, Evgeniy Zavyalov, Maya Dymova, Vladimir Richter, Elena Kuligina

**Affiliations:** 1 Institute of Chemical Biology and Fundamental Medicine Siberian Branch of the Russian Academy of Sciences, Akad. Lavrentiev Ave, 8, Novosibirsk 630090, Russia; a.ageenko@alumni.nsu.ru (A.A.); senkova_av@1bio.ru (A.S.); dymova@1bio.ru (M.D.); richter@niboch.nsc.ru (V.R.); kuligina@niboch.nsc.ru (E.K.); 2 LLC “Oncostar”, Inzhenernaya Str 23, Novosibirsk 630090, Russia; 3 Federal Research and Clinical Center of the Federal Medical and Biological Agency of Russia, Malaya Pirogovskaya Str 1a, Moscow 119435, Russia; yusubalieva.gm@fccps.ru (G.Y.); zabozlaev.fg@fnkc-fmba.ru (F.Z.); 4 Federal Research Center Institute of Cytology and Genetics, Siberian Branch of the Russian Academy of Sciences, Akad. Lavrentiev Ave, 10, Novosibirsk 630090, Russia; arom@bionet.nsc.ru (A.R.); zavjalov@bionet.nsc.ru (E.Z.); 5 Department of Medical Nanobiotechnology, Pirogov Russian National Research Medical University, Ministry of Health of the Russian Federation, Ostrovityanova Str 1, Moscow 117997, Russia; gubskii_il@rsmu.ru; 6 Federal Center of Brain Research and Neurotechnologies, Federal Medical Biological Agency of Russia, Ostrovityanova Str 1, Moscow 117513, Russia

**Keywords:** C6 glioma, GL261 glioma, virotherapy, oncolytic virus delivery

## Abstract

Virotherapy is a promising method for treating oncological diseases, including such aggressive and difficult-to-treat brain tumors such as glioblastoma. Recombinant vaccinia virus VV-GMCSF-Lact has previously shown high antitumor potential against tumor cells of varying histogenesis, including gliomas, and completed a Phase I clinical trial, demonstrating safety and good tolerability in patients with recurrent/refractory metastatic breast cancer. Investigating two types of VV-GMCSF-Lact delivery, intravenous and intratumoral, into orthotopically transplanted C6 glioma in rats, it was shown that intratumoral injection significantly increases tumor volumes in comparison with intravenous virus delivery and is accompanied by noticeable toxic effects. Extensive areas of necrotic decay of tumor tissue and its significant mixed-cell infiltration and peritumoral edema, affecting the tumor volume, were detected using H&E staining of C6 tumors after intratumoral injection of VV-GMCSF-Lact. However, only with intratumoral administration was a significant decrease in the level of the tumor cell proliferation marker Ki67 demonstrated by immunohistochemical staining. The observed toxic effects of VV-GMCSF-Lact with intratumoral administration revealed the need for dose selection, which was performed on a mouse GL261 glioma model. Results of the study allowed us to determine the viral dose that does not lead to toxic effects and can potentially increase life expectancy of mice. The data obtained show the need for careful selection of both the route of viral drug dose and administration.

## 1. Introduction

Glioblastoma (GBM) is one of the most common and aggressive tumors of the central nervous system. The median overall survival of patients with this diagnosis remains about 15 months despite standard treatment, which includes surgery and chemo- and radiotherapy [1]. Developing successful approaches for glioma treatment requires careful choice of in vivo models for further preclinical trials. Modeling of malignant neoplasms in vivo offers numerous advantages over in vitro experiments due to studying important aspects of glioma biology, such as angiogenesis, invasion, and metastasis, which cannot be studied in cell cultures [2]. Currently, the most frequently used in vivo models for glioma investigation are xenografts, which are obtained by transplantation of cells or individual human biopsies into the brain of immunodeficient animals, e.g., mice or rats, that do not reject human cells [3]. In contrast to xenograft, syngeneic models use immunocompetent animals, which makes it possible to study the interactions between the tumor and the immune microenvironment [4]. Among them, the most interesting syngeneic allografts of glioma are created by transplanting a culture of tumor cells into an organism of a species with the same genetic background [5]. Thus, after orthotopic transplantation of allogeneic tumor cells, cellular and humoral immune responses can occur, which is an advantage in the study of immunotherapeutic drugs against brain tumors [6].

One commonly used allogeneic model of glioblastoma is C6 rat glioma, which was induced by injecting N-Nitroso-N-methylurea into the brains of neonatal rats [7]. The resulting tumors largely duplicate the characteristics of human glioblastoma, such as nuclear polymorphism, high mitotic index, foci of tumor necrosis, intratumoral hemorrhages, and parenchymal invasions [8]. It should be noted that, genetically, the cells have a wild-type p53 gene, increased Rb gene expression, decreased expression of IGF-2, FGF-9, and FGF-10, and a mutant p16/Cdkn2a/Ink4a locus but do not express p16 and p19ARF mRNA [9]. On the other side, C6 cells overexpress the same genes that are expressed in human gliomas: platelet-derived growth factor subunit β, IGF-1, epidermal growth factor receptor, and Erb3/Her3, TGFα precursor proteins. They also exhibit upregulation of the Ras pathway. Thereby, C6 glioma can form a highly angiogenic, invasive tumor that mimics many morphologic characteristics of human glioblastoma, such as tumor growth, invasion and migration, angiogenesis, blood–brain barrier disruption, and production and regulation of growth factors [8]. The above properties make C6 glioma a relevant model for studying new therapeutic approaches in therapy of malignant tumors. In addition to C6, rat models such as gliosarcoma 9L and glioma F98, whose molecular, histological, and immunological characteristics have already been well described, are also widely used [10,11].

Murine glioma GL261 cells are widely used as a syngeneic animal model, which was generated by chemical induction with methylcholanthrene [12]. The cells have the following molecular and genetic features: wild-type Idh1/2, point mutations in the K-ras and p53 genes, resulting in high expression of c-myc [13]. Due to PTEN deficiency in GL261 cells, GL261 tumors closely model the PI3K pathway dysregulation that promotes glial tumor development. Recently, it has been shown that the GL261 mouse model more closely resembles developing tumors (Richards subtypes of glioblastoma), with the composition of the immunosuppressive tumor-immune microenvironment (TIME) in GL261 more closely resembling human GBM. The mouse TIME shares key gene programs characteristic of human GBM, including TNF, IFN, and hypoxia. GL261 cells exhibited basal levels of major histocompatibility complex (MHC) I, so GL261 tumors are partially immunogenic [14]. GL261 cell lines also exhibit reduced expression of MHC II and B7-1, as well as B7-2, which contributes to their evasion of immune surveillance. Tumors derived from GL261 cells recapitulate many of the characteristics of GBM, such as four-stage tumor formation: perivascular organization, proliferation near the vasculature, hypoxia via degeneration of blood vessels, and neovascularization toward necrotic areas [15]; histological, morphological features such as pleomorphism, pseudopalisading necrosis, and angiogenesis [16]. Thus, the GL261 brain tumor model is the best characterized syngeneic immunocompetent model that can be effectively used to study the antitumor effects of various therapeutic methods, but the moderate immunogenicity of the cells should be taken into account. In addition to the mouse models mentioned, there are other glioma models: GL26, CT-2A, SMA-560, and 4C8, each of which is characterized by its own molecular and histological features, but in general they all represent a suitable platform for testing new immunotherapeutic approaches [15]. In recent years, various new glioblastoma models have been developed. In most cases, this has been performed using viral vectors that have either been used to manipulate isolated mouse cells (mGB2, NSCL61, and bTiTs-G3) or injected directly into the brain of the animals (SB28, 005 GSC, and NFpp10). Besides this, tumor models have been generated from spontaneously developing tumors in genetically altered mice (KR158B and Mut3) or by culturing old cell lines in a different way (CT-2A) [17]. The abovementioned cell models have different sensitivities to existing therapy (radiotherapy, temozolomide, immunotherapy), but the decision to use a particular cell model is best made based on a comprehensive analysis of all factors, from morphological features to the molecular-genetic landscape.

Therapy by oncolytic viruses is one of the promising approaches for cancer treatment, including glioma. Virotherapy is an immunotherapeutic method that also induces an immune response, which is due to the fact that tumor cells die both as a result of direct lysis and indirectly as a result of the induction of systemic antitumor immunity [18]. This approach requires immunocompetent models to investigate interactions between an oncolytic virus and the immune system of an organism [19]. To date, different oncolytic viruses are in various stages of clinical trials against glioblastoma (DNX-2401, G207, PVSRIPO, REOLYSIN, etc. (https://clinicaltrials.gov/, accessed on 30 July 2025)) [20]. The first oncolytic virus for the therapy of human glioblastoma, Delytact (G47∆, Teserpaturev), based on a strain of herpes simplex virus type 1, was approved for use in 2021 in Japan. G47∆ demonstrated a survival benefit and safety profile in patients with progressive glioblastoma [20]. Previously, we have developed a recombinant strain of the vaccinia virus VV-GMCSF-Lact, which contains deletions of viral thymidine kinase (*tk*) and virus growth factor (*vgf*) gene fragments, in the regions of which genes of human granulocyte–macrophage colony-stimulating factor (GMCSF, *CSF2*) and the oncotoxic peptide lactaptin, a fragment of human kappa-casein (*CSN3*), were inserted, respectively. Deletions in *tk* and *vgf* genes increase the selectivity of the virus against tumor cells and decrease the virulence for normal cells. The GMCSF expression stimulates the antitumor immune response. The choice of lactaptin as a transgene is due to the fact that this peptide induces tumor cell apoptosis via both the receptor-mediated and mitochondrial pathways [21,22,23]. The action of lactaptin leads to a decrease in the level of antiapoptotic protein Bcl-2 and an increase in the level of proapoptotic protein Bax, phosphatidylserine translocation to the outer surface of the cytoplasmic membrane, a change in the transmembrane potential of mitochondria and activation of caspase-3 and caspase-7, as well as oligonucleosomal fragmentation of DNA [23,24,25,26]. In previous studies we have shown that VV-GMCSF-Lact has high cytotoxic activity against a wide panel of cancer cell cultures of varying histogenesis, including triple negative breast cancer, lung cancer, epidermoid carcinoma, and glioblastoma [24,27]. In addition, the antitumor efficacy of the virus against human glioblastoma xenografts in immunodeficient mice has been shown [15]. Preclinical studies of the double recombinant vaccinia virus VV-GMCSF-Lact against breast cancer was completed in 2019 and recently the safety and good tolerability of the virus drug were confirmed in a Phase I clinical trial in patients with recurrent/refractory metastatic breast cancer with single and multiple administration (NCT05376527).

The purpose of this work is to investigate the therapeutic efficacy of VV-GMCSF-Lact against rat C6 and murine GL261 gliomas. We have shown that VV-GMCSF-Lact has high cytotoxic activity against C6 and GL261 glioma cells in vitro. When evaluating the antitumor efficacy of the virus against orthotopically transplanted C6 glioma in vivo, we showed that intratumoral administration of the virus did not affect the volume of primary tumors, compared with the intravenous administration group, however, it completely blocked the formation of distant foci of invasive glioma growth in experiments on rats. We also assessed the impact of different doses of the virus on its antitumor efficacy, and in experiments on mice identified the dose that can be effective and safe for the treatment of gliomas when the drug is administered intratumorally. Moreover, the lifespan of mice in the highest dose group was maximal and reached 160 days since the treatment initiation. Our data revealed that VV-GMCSF-Lact is a promising antitumor agent for the treatment of glial tumors.

## 2. Materials and Methods

### 2.1. Glioma Cell Cultivation

Rat C6 glioma cells, received from the Collection of Cell Cultures of the Federal Research and Clinical Center of the Federal Medical and Biological Agency of Russia, Moscow, Russia, were cultured in IMDM medium (Thermo Fisher Scientific, Waltham, MA, USA) supplemented with 10% FBS (Thermo Fisher Scientific, Waltham, MA, USA), 2 mM L-glutamine (Invitrogen, Waltham, MA, USA), and antimycotic/antibiotics solution (100 U/mL penicillin, 100 mg/mL streptomycin sulfate, 0.25 μg/mL amphotericin) (Sigma-Aldrich, Darmstadt, Germany) in culture flasks with surface area 25 cm^2^ (TPP, Trasadingen, Switzerland).

Mouse GL261 glioma cells, kindly provided by Alexey Stepanenko (Department of Neurobiology, V. P. Serbsky National Medical Research Center for Psychiatry and Narcology) were cultured in DMEM/F12 medium (Thermo Fisher Scientific, Waltham, MA, USA) supplemented with 20% FBS (Thermo Fisher Scientific, Waltham, MA, USA), 2 mM L-glutamine (Invitrogen, Waltham, MA, USA), and antimycotic/antibiotics solution (100 U/mL penicillin, 100 mg/mL streptomycin sulfate, 0.25 μg/mL amphotericin) (Sigma-Aldrich, Darmstadt, Germany) in culture flasks with surface area 25 cm^2^ (TPP, Trasadingen, Switzerland).

Both glioma cell cultures (rat C6 and mouse GL261) were used to model experimental orthotopic glioma in Wistar rats and C57Bl/6 mice, respectively. Cell counting was performed in a Countess 3 automated cell counter (Thermo Fisher Scientific, Waltham, MA, USA). Immediately before implantation into rodents, glioma cells were detached from the culture dish using TrypLE Express Enzyme (Thermo Fisher Scientific, Waltham, MA, USA). The harvested cells were washed from the medium with TrypLE and re-suspended in sterile Dulbecco’s phosphate–salt buffer (DPBS) solution for subsequent injection.

### 2.2. Evaluation of the VV-GMCSF-Lact Cytotoxic Activity Against Glioma Cells In Vitro

The cytotoxic VV-GMCSF-Lact activity against rat C6 and mouse GL261 glioma cells was performed using the standard MTT test. For this, cells were seeded into 96-well culture plates (TPP, Switzerland) in an amount of 4000 cells per well.

To determine CD50 (cytotoxic dose causing death of 50% of cells), VV-GMCSF-Lact was added to cells in 100 μL of RPMI medium (Thermo Fisher Scientific, Waltham, MA, USA) to a final virus concentration in culture medium from 0.00012 to 10 PFU per cell. The plates were incubated for 72 h at 37 °C in 5% CO_2_. Control cells were incubated under the same conditions without the virus. After incubation, the culture medium was removed from the wells, 200 μL of MTT reagent (Applichem, Darmstadt, Germany) diluted in RPMI medium (up to 0.5 mg/mL) was added to each well, and the cells were incubated for 4 h at 37 °C. Then the medium with MTT was removed and the formed formazan crystals were dissolved in 150 μL DMSO (MP Biomedicals, Irvine, CA, USA). The optical density of the formazan solution was measured at a wavelength of 570 nm on an Apollo LB 912 spectrophotometer (Berthold Technologies, Bad-Wildbad, Germany). Cell viability was determined relative to control cell viability (100%) ± standard deviation from three independent experiments. CD50 was calculated using CompuSyn software, version 1.4 (ComboSyn, Inc., Paramus, NJ, USA).

### 2.3. Experimental Animals

The study was carried out in compliance with the ARRIVE guidelines. All experiments with laboratory animals were carried out in accordance with the recommendations and requirements of the World Society for the Protection of Animals (WSPA) and the European Convention for the Protection of Vertebrate Animals (Strasbourg, 1986). The protocols were approved by the Ethics Committee of the Federal Research and Clinical Center of the Federal Medical and Biological Agency of Russia (Protocol No. 7 dated 6 September 2022).

The experiment of analyzing two ways of VV-GMCSF-Lact administration was performed on 23 adult female Wistar rats obtained from the Stolbovaya branch of the Federal State Budgetary Scientific Institution “National Center for Biomedical Technologies of the Federal Medical and Biological Agency of Russia”, weighing 200–220 g. Rats were housed in groups in conventional cages with a 12/12 h light/dark cycle, a temperature of 20–24 °C, and a humidity of 45–55%. Water and standard chow were provided ad libitum.

The experiment of optimizing the therapeutic dose of VV-GMCSF-Lact was performed on 40 adult female C57BL/6 mice obtained from the Stolbovaya branch of the Federal State Budgetary Scientific Institution “National Center for Biomedical Technologies of the Federal Medical and Biological Agency of Russia”, weighing 18–20 g. Mice were housed in groups in conventional cages with a 12/12 h light/dark cycle, a temperature of 20–24 °C, and a humidity of 45–55%. Water and standard chow were provided ad libitum.

All manipulations with animals were carried out at the Pirogov Russian National Research Medical University, Ministry of Health of the Russian Federation, Moscow.

### 2.4. Orthotopic Transplantation of Glioma Cells into Immunocompetent Animals

To model orthotopic allogeneic glioma, C6 glioma cells were transplanted into the striatum region (coordinates Ap –1, L 3.0, V 4.5 according to the rat brain atlas edited by Swanson (http://larrywswanson.com/) accessed on 20 June 2025) in the amount of 500,000 cells per animal. Before the start of the surgical intervention, the animals were injected intraperitoneally with an aqueous solution of Telazol (Zoetis, Parsippany, NJ, USA) in a dose of 0.08–0.12 mL. After the onset of the stage of anesthesia, the scalp was cleaned from hair and the animal was fixed on a stereotaxic system (Leica Biosystems, Danvers, MA, USA). The scalp was disinfected with 70% ethanol and an incision was made in the soft tissues of the head along the sagittal suture between the fronto-parietal and parieto-occipital sutures using a scalpel. The scalp was pushed apart and fixed with a surgical retractor, after which the bone was skeletonized. The manipulator of the stereotaxic system was adjusted to the coordinates Ap: −1, L: 3.0 to determine the point at which a burr hole was subsequently made using a dental drill. Through this hole, using a Hamilton syringe (Hamilton Company, Reno, NV, USA) connected to a manipulator and an infusomat, the cell suspension was injected at a rate of 3 μL/min, with the needle previously positioned in the V: 4.5 coordinate of the stereotaxic coordinate system. At the end of the administration of the cell suspension, the needle was left in the hole for 5 min. Then the needle was carefully removed, the soft tissues were sutured with interrupted sutures, and the wound surface was treated. The development of tumors was monitored using MRI scanning on a Biospin MRI CLINSCAN (Bruker, Billerica, MA, USA).

To model orthotopic syngeneic glioma, 4- to 5-week-old mice were anaesthetized with Zoletil (40 mg/kg, 50 μL, Virbac SA, Carrosse, France) and Romethar (50 μL, Interchemie, Limburg, The Netherlands) and fixed through the ear canals in a stereotaxic system (RWD Life Science, Shenzhen, China). Mice were scalped and a trepanation hole was made in the skull in the projection of the striatum of the right cerebral hemisphere using Drill Bits HM1005 0.5 mm, Round Tip (RWD Life Science, Shenzhen, China). GL261 cells (100,000 cells in 5 μL of sterile Dulbecco’s phosphate–salt buffer) were injected using a micropump at a rate of 1 μL/min. A suspension of glioma GL261 cells was stereotactically injected into the brain striatum: A-1+ D1,5 V-2,5 mm.

### 2.5. Evaluation of the VV-GMCSF-Lact Antitumor Efficacy Against Orthotopically Transplanted Immunocompetent Glioma

#### 2.5.1. Analysis of the Therapeutic Efficacy of Intravenous and Intratumoral VV-GMCSF-Lact Administration

Twenty-three female Wistar rats with orthotopically transplanted and MRI-confirmed C6 tumors were randomly divided into three groups: nine animals each in the intratumoral and intravenous groups and five rats in the control group, using the = RAND() function (Microsoft Excel version 16, Redmond, WA, USA) (Table 1). Each rat, therefore, was considered to be an experimental unit. The sample size was calculated using online software (Lamorte’s Power Calculations, https://www.bu.edu/research/forms-policies/iacuc-sample-size-calculations/, accessed on 20 June 2025). Intact controls were also used: four healthy non-tumor-bearing rats, which were injected with VV-GMCSF-Lact intravenously (two rats) or intracerebrally (two rats) once in the same dose as the tumor-bearing rats from each experimental group. Intracerebral VV-GMCSF-Lact injections were performed using the same stereotaxic coordinates as for the intratumoral injections.

Investigators involved in administering the viral drug, performing MRI scans, and monitoring the animals during the experiment could not be blinded to whether the animal were injected intravenously or intratumorally because of typical lesions due to each type of drug administration. The investigator who performed the histology and immunohistochemistry analysis of rat brains was blinded about the treatment group allocation.

All surgical interventions were performed on animals anaesthetized with Zoletil (Virbac SA, Carrosse, France) and Domitor (Orion Corporation, Espoo, Finland). Zoletil was used for anesthesia and myorelaxation, and Domitor was used for additional sedation of experimental animals. Intratumoral administration of virus was performed using a stereotaxic system using coordinates (Ap –1, L 3.0, V 4.5 according to the rat brain atlas edited by Swanson (http://larrywswanson.com/) accessed on 20 June 2025) determined from MRI images on the 6th day after tumor cell transplantation. In this case, the virus was applied in fractions to different locations within the tumor by gradually reducing the depth of injection from the most basal tumor margin visualized by MRI, changing the injection angle. VV-GMCSF-Lact was injected in a dose of 1.65 × 10^7^ PFU/50 μL per rat. When administered intravenously, the drug was injected into animals through the inferior vena cava in a dose of 3 × 10^8^ PFU/1 mL per rat. Animals from the control group did not receive any injections.

On the 13th, 20th, and 26th days after the transplantation of tumor cells (on the 6th day after each drug injection) MRI scanning of tumors was performed in the T2 and T1 modes with contrast enhancement using a gadolinium-containing contrast agent Omniscan (GE Healthcare, Chicago, IL, USA).

#### 2.5.2. Optimizing the Therapeutic Dose of VV-GMCSF-Lact

Forty female C57BL/6 mice in which the tumor node on day 8 after orthotopical glioma cell transplantation was detected by MRI were taken into the experiment and were divided into 4 groups, 10 mice in each group, so that the average tumor volume is the same and is approximately 0.23–0.33 mm^3^ (Table 2). Each mouse, therefore, was considered to be an experimental unit. One mouse did not develop a tumor after transplantation and was not included in any experimental group. The sample size was calculated using online software (Lamorte’s Power Calculations).

Investigators who prepared oncolytic virus for intratumoral injection and controlled the process of each virus dose administration to the appropriate experimental group and the investigator who was responsible for anesthesia and surgical procedures were aware about the animal allocation; the investigator who performed the MRI scanning and its analysis and the investigator who performed the histology and immunohistochemistry analysis were blinded about animal group allocation.

All surgical interventions were performed on animals anesthetized with Zoletil (Virbac SA, Carrosse, France) and Domitor (Orion Corporation, Espoo, Finland). Zoletil was used for anesthesia and myorelaxation, and Domitor was used for additional sedation of experimental animals. Intratumoral administration of virus was performed using a stereotaxic system using coordinates A-1+ D1,5 V-2,5 mm.

The primary outcome of this study is assessment of tumor-bearing rat survival and tumor size when the viral drug is administered intratumorally or intravenously. Secondary outcomes include the development/absence of secondary tumor foci and assessment of morphological changes in tumor tissue.

To evaluate VV-GMCSF-Lact efficacy, tumor size, mean survival time, and increase in lifespan were estimated.

The tumor volumes were calculated using image processing and analysis program MicroDicom DICOM Viewer, 32-bit version (MicroDicom Ltd., Sofia, Bulgaria) using the following equation:

V = ΣS_t_ × (D_s_ + T_s_),

where ΣS_t_—the sum of slice areas; D_s_—the slice distance; T_s_—the slice thickness.

### 2.6. MRI Study

#### 2.6.1. Rats

Xenotransplanted tumor characterization was performed by the 7T ClinScan (Bruker, Billerica, MA, USA) system for small animals, software version Syngo VBA15A_Update_6.0. A rat brain array surface multichannel coil was used with entire brain coverage. MRI scanning and subsequent processing of the resulting images were carried out in accordance with the following protocol. Two to three minutes before the study, rats were immobilized with 4% isoflurane (Baxter Healthcare Corp., Deerfield, IL, USA) using an anesthesia machine in an air flow mode of 300–350 mL/min (The Univentor 400 Anaesthesia Unit, Univentor, Zejtun, Malta). Anesthesia was also maintained during MRI scans, with only 2% anesthesia delivered. The temperature of the animals was maintained using a water circuit in a tomography stage, which had a surface temperature of 30 °C. A pneumatic breathing sensor (SA Instruments, Stony Brook, NY, USA) was placed under the lower body to monitor the depth of anesthesia. The MR protocol includes:

1. T2-weighted image in transversal projection (2D Turbo Spin Echo with restore magnetization pulse; turbo factor = 10; TR/TE = 6300/46 ms; flip angle = 160; averages = 2; spectral fat saturation; FOV = 30 × 21 mm; slice thickness = 0.7 mm; matrix size = 256 × 144);
2. T2-weighted image in coronal projection (2D Turbo Spin Echo with restore magnetization pulse; turbo factor = 10; TR/TE = 4800/46 ms; flip angle = 160; averages = 2; spectral fat saturation; FOV = 32 × 22 mm; slice thickness = 0.7 mm; matrix size = 256 × 144);
3. T2-weighted image in sagittal projection (2D Turbo Spin Echo with restore magnetization pulse; turbo factor = 10; TR/TE = 5400/46 ms; flip angle = 160; averages = 2; spectral fat saturation; FOV = 32 × 22 mm; slice thickness = 0.7 mm; matrix size = 256 × 144);
4. DWI (with ADC calculation on MR system software, version Syngo VBA15A_Update_6.0)—2D diffusion-weighted image with calculation of apparent diffusion coefficient maps (TR/TE = 9000/33 ms; flip angle = 90; 14 b factors = from 0 to 2000 s/mm^2^; averages = 1; spectral fat saturation; FOV = 32 × 20 mm; slice thickness = 1.0 mm; matrix size = 80 × 52);
5. T1-weighted image in transversal projection with gadolinium contrast agent (2D Turbo Spin Echo; turbo factor = 3; TR/TE = 540/9.9 ms; flip angle = 160; averages = 2; spectral fat saturation; FOV = 30 × 21 mm; slice thickness = 0.7 mm; matrix size = 256 × 144; gadodiamide, Omniscan ^®^ (GE Healthcare, Chicago, IL, USA), 40 µmole/rat).

#### 2.6.2. Mice

MR examination was performed with the 7T ClinScan (Bruker BioSpin) system, software version Syngo VBA15A_Update_6.0. A mouse brain array volume coil was used with entire brain coverage. The MR protocol includes:

T2-weighted image in transversal projection (2D Turbo Spin Echo with restore magnetization pulse; turbo factor = 14; TR/TE = 3310/46 ms; flip angle = 140; averages = 2; spectral fat saturation; FOV = 14 × 19 mm; slice thickness = 0.5 mm; matrix size = 260 × 384);

T2-weighted image in coronal projection (2D Turbo Spin Echo with restore magnetization pulse; turbo factor = 18; TR/TE = 2500/46 ms; flip angle = 140; averages = 2; spectral fat saturation; FOV = 14 × 19 mm; slice thickness = 0.5 mm; matrix size = 260 × 384);

T2-weighted image in sagittal projection (2D Turbo Spin Echo with restore magnetization pulse; turbo factor = 18; TR/TE = 3000/47 ms; flip angle = 140; averages = 2; spectral fat saturation; FOV = 14 × 19 mm; slice thickness = 0.5 mm; matrix size = 260 × 384);

Morphometry (quantitative assessment of volume) of the tumor was performed using the MicroDicom DICOM Viewer (MicroDicom Ltd., Bulgaria) on T2-weighted images. Initially, the area of damage was measured separately on each slice, and then the volume of the entire tumor was calculated using the formula: V = (S1 + … + Sn) × (h + d), where V is the volume, S1 … n is the measured area on each slice, h is the slice thickness, and d is the interslice gap. The tumor growth rate (TGR) was calculated based on T2-weighted MRI data using the formula:

TGR = (V_max_ − V_t_)/(Δt × V_max_),

where V_t_—the tumor volume right after first injection, V_max_—maximal detected tumor volume, Δt—time required to reach V_max_.

### 2.7. Histology and Morphometry

The rat and mouse brains were cut in the coronal (also called frontal) plane for subsequent histology. For the histological study, the brain specimens with tumor nodes were fixed in 10% neutral-buffered formalin (BioVitrum, Moscow, Russia), dehydrated in ascending ethanol and xylol series, and embedded in HISTOMIX paraffin (BioVitrum, Saint-Petersburg, Russia). The paraffin sections (up to 5 µm) were sliced on a Microm HM 355S microtome (Thermo Fisher Scientific, Waltham, MA, USA) and stained with hematoxylin and eosin. The images were examined and scanned using an Axiostar Plus microscope equipped with an Axiocam MRc5 digital camera (Zeiss, Oberkochen, Germany) at magnifications of ×100, ×200, and ×400.

Morphometric analysis of tumor nodes was performed using a counting grid consisting of 100 testing points in a testing area equal to 3.2 × 10^6^ μm^2^ and included evaluation of the volume densities (Vv, %) of unaltered tumor tissue, cell infiltration, necrosis and hemorrhages in the tumor foci, as well as evaluation of the numerical densities (Nv) of mitoses in the tumor tissue. Five to ten random fields, from the tumor specimens of three to five mice in each group (total of 15–50 testing fields), were studied.

### 2.8. Immunohistochemistry of Rat C6 Glioma

Immunohistochemical studies were carried out on sections 3–4 µm thick from ready-made paraffin blocks containing the tissue samples under study using the polymer–protein–peroxidase method according to standard methods using an imaging system and antibody concentrates [28]. Monoclonal rabbit antibodies against Ki-67 (30-9) and GFAP (EP 672Y) (Ventana, Oro Valley, AZ, USA) were used as primary antibodies. Working solutions diluted 1:100 were prepared from lyophilized concentrates prediluted in 100 μL of AntiBody Diluent. After deparaffinization and dehydration in order to block endogenous peroxidase, the sections were treated with 0.3% H_2_O_2_ for 20 min, washed in distilled water, and, in order to unmask antigenic determinants, were subjected to heat treatment in a buffer solution for 30 min t = 98 °C. After washing in a buffer solution 3 times for 5 min each, working dilutions of primary antibody concentrates were applied. Incubation with primary antibodies was carried out for 30 min at room temperature. After incubation with primary antibodies, sections were washed in a washing buffer solution previously prepared from the concentrate 3 times for 5 min each, then a polymer–protein–peroxidase complex detection system was applied for 30 min at room temperature. Detection of antigenic epitopes was carried out using DAB. The study was carried out using the VENTANA BenchMark ULTRA immunostainer (Ventana, Oro Valley, AZ, USA).

The assessment of the immunohistochemical reaction with determination of the level of cell proliferation was carried out in the form of the percentage of volume occupied by positive cells with nuclear Ki-67 (30-9), as well as by the semi-quantitative method of GFAP-positive cells (EP 672Y) in the area of brain damage in the growth area tumor and in its marginal zones. Counting was carried out at a magnification of ×100, and the abovementioned volume occupied by Ki-67-positive nuclei of cellular structures was calculated.

### 2.9. Statistical Analysis

The rodent survival data were analyzed by Kaplan–Meier survival analysis. The Mann–Whitney U test, also known as the Wilcoxon rank sum test, was used to compare two samples or groups. A *p*-value of <0.05 was considered statistically significant in all instances. The normalized Pearson’s chi-square test was used for the estimation of additional tumor site distribution. All error bars represent standard deviation of the mean.

## 3. Results

### 3.1. Cytotoxic Activity of VV-GMCSF-Lact

**Figure 1:**
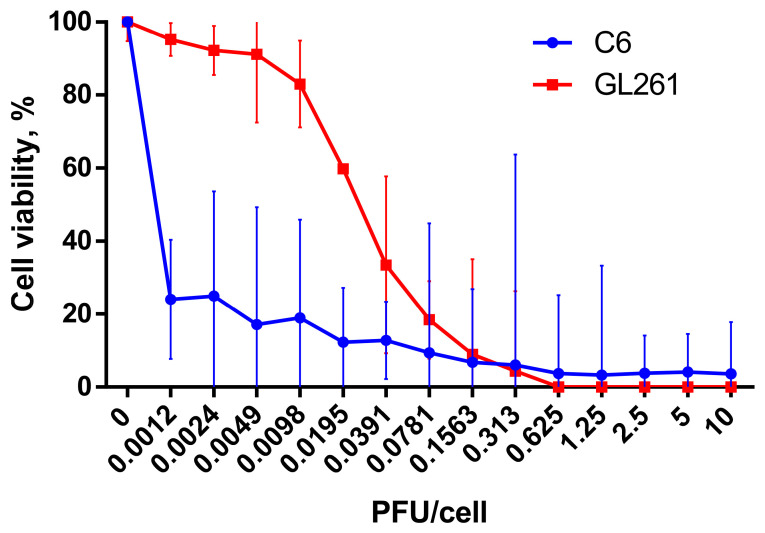
Cell viability of C6 (blue curve) and GL261 (red curve) glioma cells under the action of VV-GMCSF-Lact. Cells were infected with the recombinant virus (0.0012–10 PFU/cell) and incubated for 72 h. The 50% cytotoxic dose (CD50) was evaluated using MTT test. The results are presented as a mean value of percentage of control ± 95% CI from three independent experiments.

Confirming the prospect of using C6 and GL261 gliomas as models for investigation of antitumor efficacy of oncolytic virus in immunocompetent animals in vivo, we evaluated cytotoxic activity of VV-GMCSF-Lact against rat C6 and Gl261 glioma cells in vitro. VV-GMCSF-Lact effectively reduced the viability of C6 and GL261 cells and the CD50 of the oncolytic virus was 0.004 PFU and 0.0248 PFU per cell, respectively (Figure 1).

### 3.2. Antitumor Efficacy of VV-GMCSF-Lact Against Rat C6 Allograft

**Figure 2:**
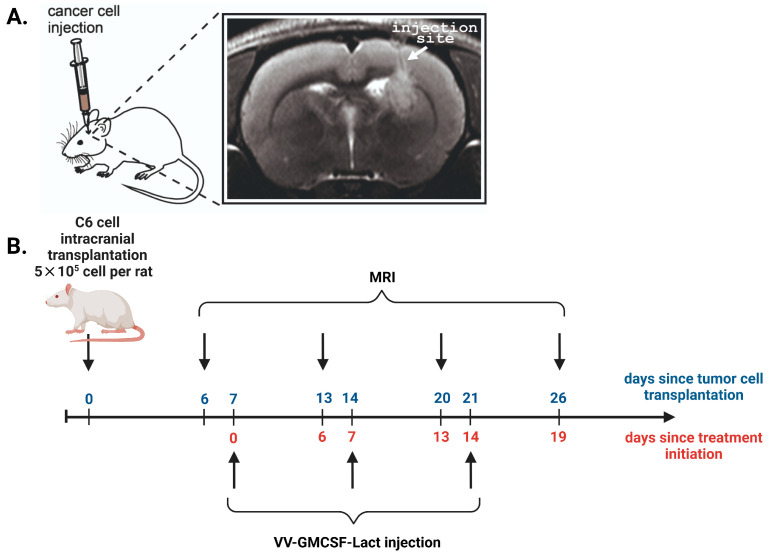
(**A**) MRI localization of the injection site of C6 glioma cells into the rat brain. In T2-weighted images, white arrows indicate the site of cancer cell inoculation into the striatum region (coordinates Ap –1, L 3.0, V 4.5 according to the rat brain atlas edited by Swanson). T2-hyperintense lesion (tumor) is visualized in upper part of striatum, periventricular to lateral ventricle with growth along injection track through corpus callosum. (**B**) The scheme of the experiment showing days since the start of two time points: the tumor cell transplantation (blue) and the initiation of C6 glioma treatment (red).

VV-GMCSF-Lact antitumor efficacy against rat C6 glioma was assessed in immunocompetent female Wistar rats with orthotopically transplanted tumors (Figure 2A).

To estimate the dynamics of tumor growth, T2-weighted MRIs were performed 6–26 days after C6 glioma intracranial injection. On the 6th day after the tumor cell transplantation, the tumor formation was detected in all experimental animals with average tumor size of about 1.3 mm^3^. Then the animals were randomly divided into three experimental groups: group A—intratumoral administration (i.t.); group B—intravenous administration (i.v.); group C—control group (Table 1). Treatment was started on the 7th day after tumor cell transplantation according to the developed scheme for each experimental group (Figure 2B).

**Figure 3:**
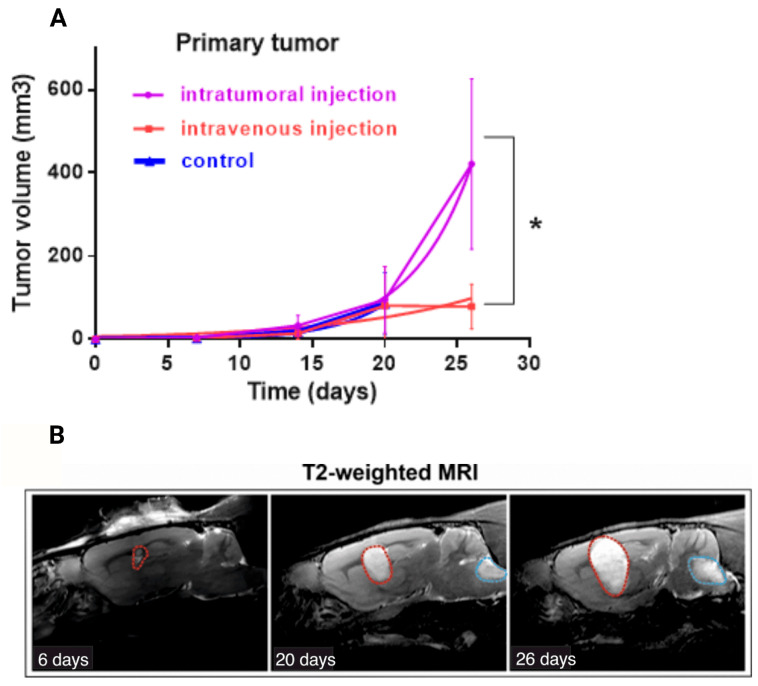
(**A**) Effect of intratumoral and intravenous administration of VV-GMCSF-Lact on the primary tumor volumes of allograft C6 glioblastoma (doubling time method). Values are mean ± SD of nine independent experiments *p* ≤ 0.05 (*) (**B**) Example of the time course (6–26 days after cell injection) of the C6 primary tumor (red dotted line) and secondary tumor site (blue dotted line) growth in vivo. T2-weighted MRI was used to visualize the tumor.

The dynamics of tumor growth/regression were estimated using MRI visualization (Figure 3A). Analysis of MRI data showed that all control animals and seven of nine animals that were administered the drug intravenously showed the appearance of secondary tumor foci from the 13th day after the start of treatment (the 20th day after the tumor cell transplantation) (Figure 3B, Table 3). Moreover, in animals in the group with intratumoral administration of VV-GMCSF-Lact, secondary nodes were not observed during the experiment.

A doubling time graph was constructed by fitting the exponential growth model to our data. Doubling time for the intratumoral administration group was 2930, for the intravenous administration group—6670, for the control group—2557, which indicates greater efficiency of the virus when administered intravenously. Although, the tumor volume for the group of intratumoral administration was greater than for the group with intravenous administration (*p* < 0.05) (Figure 3A), no rats from the intratumoral administration group developed additional tumor sites (Table 3). We obtained statistically significant differences for the occurrence of additional tumor sites between the i.t. and i.v. groups on the 20th day since the tumor cell transplantation (*p* < 0.05, normalized value of Pearson’s coefficient (C’) was 0.826), and on the 26th day of observation we have *p* > 0.05, although the normalized Pearson coefficient (C’) value suggested a strong correlation (0.866) (Table 3).

**Figure 4:**
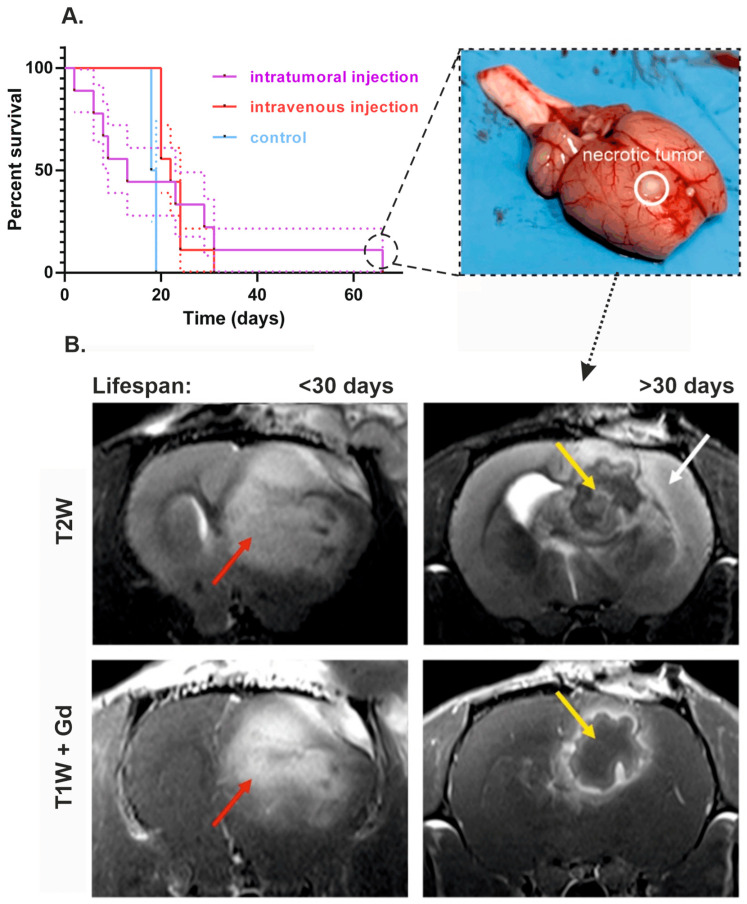
The effect of intratumoral and intravenous administration of VV-GMCSF-Lact on the survival of rats with intracranial xenotransplantation of C6 glioma cells. (**A**) Cumulative survival curves with SE (Kaplan–Meier method) of control animals, rats that received VV-GMCSF-Lact intratumorally or intravenously. The photo on the right shows an example of a rat brain whose survival rate was more than 2 times higher than the average for all groups. The solid line marks the site of injection of cancer cells and necrotic site detection. (**B**) MRI comparison of animals with lifespan less than 30 days and rat that received VV-GMCSF-Lact intratumorally and whose survival was more than 30 days. In the first animal, a T2-hyperintense lesion is visualized, diffusely accumulating MR contrast agent (red arrow). In the second animal, in the area of tumor cell transplantation, a structure with a thick wall with accumulation of contrast agent and a site of no Gd accumulation in the center are visualized—yellow arrow. Noticeable edema is noted along the periphery, spreading mainly along the cortex—white arrow.

Despite significant differences between tumor volumes, no reliable distinctions were found in the survival rate of animals from experimental and control groups (Figure 4A, Gehan–Breslow–Wilcoxon test, *p* = 0.9998). Additionally, one rat from the intratumoral administration group with the longest lifespan (66 days since the treatment started) showed reduction of almost the entire tumor mass according to MRI scans.

T2- and T1-weighted images allow delineation of the boundaries of the tumor for morphometric analysis, and Gd-enhanced T1-weighted scans also reflect the areas with blood–brain barrier breakdown. The MR picture may be due to the formation of a lesion with a capsule at the periphery and inflammatory changes in the center, as indicated by changes in DWI, a pattern of accumulation of a contrast agent and pronounced edema of the periphery (Figure S1).

**Figure 5:**
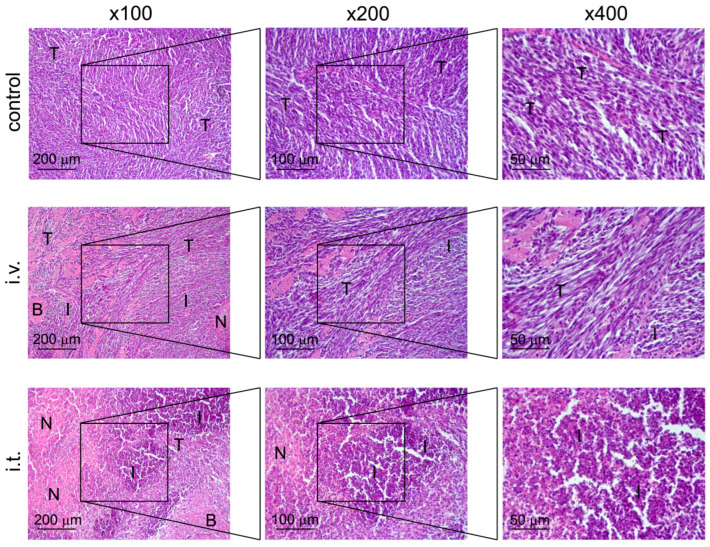
Structural changes in C6 glioma, transplanted orthotopically into the brain of rats, without treatment (control) and after administration of VV-GMCSF-Lact intravenously (i.v.) or intratumorally (i.t.). Hematoxylin and eosin staining. Original magnification ×100 (left panel), ×200 (middle panel), and ×400 (right panel). The black boxes show areas that were examined further at a higher magnification. T—unchanged tumor tissue, N—foci of necrosis, I—areas of infiltration, B—remnants of brain tissue.

The Kaplan–Meier curves demonstrate that rats from the intravenous VV-GMCSF-Lact group and the control group began to die almost at the same time—on the 16th day after the first injection of the virus (on the 23rd day after the tumor transplantation). With intratumoral administration of the virus, animals began to die on the 2nd day since the treatment started (on the 9th day after the tumor transplantation). Despite the early death of some animals from the group of intratumoral injection, it was characterized by the highest survival rate (Figure 4A). Sites of necrotic decay were noted in the group of intratumoral administration with enhanced lifespan, which were confirmed during autopsy of the animal, removal of the brain, and conducting a control section (Figure 4A and Figure 5). The formation of a lesion with a capsule and pronounced edema in the area of tumor cell transplantation was noted in rat cohort on the 6th day after the treatment started (Figure 4B). At the same time, in animals whose life expectancy was less than 30 days, on the 26th day of MRI, gadolinium was evenly distributed throughout the entire volume of the tumor, which emphasizes the solid tumor mass (Figure 4B).

### 3.3. Morphological and Immunological Study of Rat C6 Glioma Without Treatment and After VV-GMCSF-Lact Administration

Histological examination of the brain of rats with C6 glioma without treatment visualizes a tumor node with blurred boundaries and an infiltrating growth represented by undifferentiated polygonal to spindle-shaped cells forming sheets and bundles (Figure 5). The cells were characterized by centrally located irregular nuclei with 1–2 nucleoli and moderate nuclear to cytoplasmic ratio. Some cells, amounting to 2–3 per field of view, contained mitotic events. A weak mixed-cellular infiltration, represented by granulocytes and lymphocytes, located diffusely and amounting to 9 ± 2.9% from entire tumor tissue, can be detected (Figure 5, Table 4). Small foci of necrosis (3 ± 1.5%) were observed in the central areas of tumor nodes (Figure 5, Table 4).

Intravenous treatment of C6-glioma-bearing rats with VV-GMCSF-Lact led to a 3-fold increase in the volume density of cell infiltration in the tumor foci compared with the control (Figure 5, Table 4). The observed infiltration was more pronounced along the periphery of the tumor node, at the border with the brain tissue. Foci of necrotic decay were also observed in the central areas of tumor nodes and did not significantly differ from the control (4.7 ± 1.9%) (Figure 5, Table 4). Perivascular and pericellular edema in the brain tissue was detected.

As for intratumoral administration of VV-GMCSF-Lact into the C6-glioma-bearing rats, large areas of necrotic decay were identified inside the tumor nodes and amounted to 44.4 ± 14.4% from the tumor node that was 14.8 and 9.4 times more than in control and i.v. groups, respectively (Figure 5, Table 4). Massive mixed-cellular infiltration is observed inside and predominately on the periphery of tumor nodes destroying adjacent brain tissue: i.t. treatment caused 5.7- and 1.9-fold increases in the volume density of cell infiltration in the tumor tissue compared with control and i.v. treatment, respectively (Figure 5, Table 4). Perivascular and pericellular edemas were also detected.

In both groups, as with intratumoral and intravenous administration of VV-GMCSF-Lact, there was one rat out of nine with signs of thrombosis and hemorrhage in the tumor formation. With intravenous and intratumoral administration, hemorrhage foci occupied 37.7 ± 13.8% and 24 ± 3.6% of the entire tumor node, respectively (Figure S2).

It should be noted that, with administration of VV-GMCSF-Lact into the brain of healthy rats without a tumor, no destructive changes in the brain tissue were detected; only signs of slight to moderate edema and scanty cell infiltration were observed (Figure S3).

**Figure 6:**
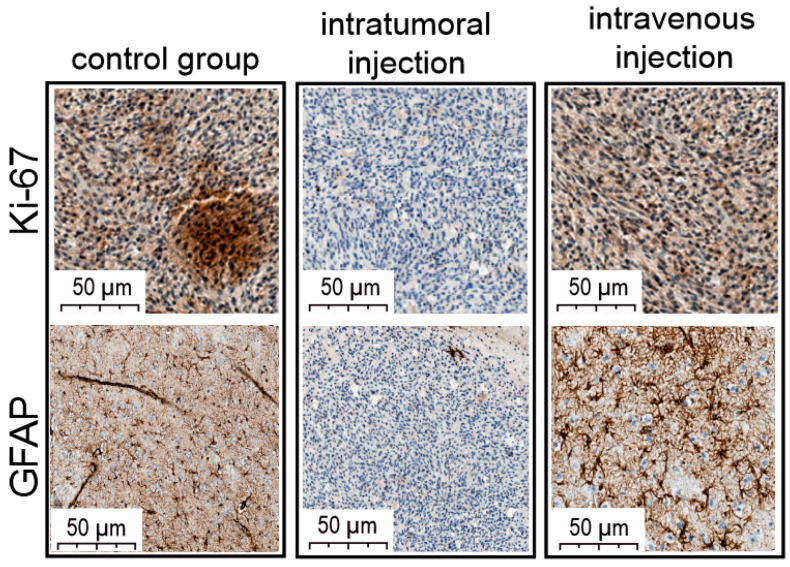
Light microscopy of antigen Kiel 67 (Antibody Ki-67) and glial fibrillary acidic protein (GFAP) immunolabeling of C6 glioma-cell-injected rat brain. The figure shows examples of brain sections from intact rats and intratumorally or intravenously VV-GMCSF-Lact-administered animals.

Notably, oncolytic properties of VV-GMCSF-Lact include antiproliferative activity, confirmed by immunohistochemical analysis of treated and non-treated tumors. To assess tumor cell proliferation during the treatment, we used the marker of proliferation Kiel 67 (Ki-67) and the glial fibrillary acidic protein (GFAP). Immunohistochemical analysis of the cell proliferation level in the tumor tissue of intravenously injected animals revealed a fairly high percentage of the immunohistochemical marker Ki-67 (Figure 6).

Also, the pronounced activity of the GFAP marker was determined with great consistency both in the central areas of tumor cell complexes and in marginal sections. As for the group of intratumoral drug administration, the Ki-67 proliferation marker of tumor cells was extremely reduced (Figure 6). The number of GFAP-positive cells in the area of brain damage, in tumor growth zones, and in marginal zones was also extremely low.

### 3.4. Antitumor Efficacy of VV-GMCSF-Lact Against GL261 Murine Glioma

**Figure 7:**
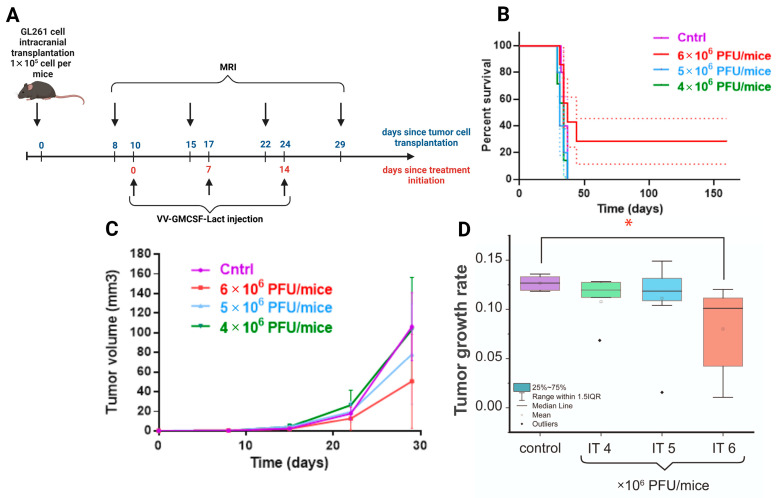
(**A**) The scheme of the experiment showing days since the start of two time points: the tumor cell transplantation (blue) and the initiation of GL261 glioma treatment (red). (**B**) Cumulative survival curves with SE (Kaplan–Meier method) of control animals and mice that received VV-GMCSF-Lact intratumorally at various doses. (**C**) Effect of different VV-GMCSF-Lact doses on the volumes of orthotopic GL261 mice glioma tumors. (**D**) Comparison of the tumor growth rate of intracranial allograft glioblastoma in mice that received VV-GMCSF-Lact in different doses (IT 4×, 5×, 6 × 10^6^ PFU/mouse). The box is drawn from Q1 to Q3 with a horizontal line drawn inside it to denote the median. *—significant intergroup differences (Mann–Whitney U test, *p* < 0.05).

Antitumor efficacy of VV-GMCSF-Lact at different doses was evaluated against GL261 murine glioma in immunocompetent female C57BL/6 mice with orthotopically transplanted tumors. To analyze the dynamics of tumor growth, T2-weighted MRIs were performed 8–29 days after intracranial injection of GL261 glioma cells. On the 7th day after the tumor cell transplantation, the tumor formation was detected with average tumor size of about 0.25 mm^3^. Mice with no forming tumors were not included in the experiment. Animals with tumors were randomly divided into four experimental groups: group D—intratumoral VV-GMCSF-Lact administration at dose 6 × 10^6^ PFU/mouse; group E—intratumoral VV-GMCSF-Lact administration at dose 5 × 10^6^ PFU/mouse; group F—intratumoral VV-GMCSF-Lact administration at dose 4 × 10^6^ PFU/mouse; group G—control group. Treatment was started on the 10th day after tumor cell transplantation according to the developed scheme for each experimental group (Figure 7A).

The changes in tumor volumes were estimated using MRI visualization (Figure 7C). According to the data obtained, no significant differences in tumor volume were observed in animals receiving VV-GMCSF-Lact at various doses.

Analysis of survival curves also does not demonstrate an increase in life expectancy, confirmed by statistical methods of analysis (Figure 7B, Gehan–Breslow–Wilcoxon test, *p* = 0.109). However, the life expectancy of two mice in the group with a high dose of VV-GMCSF-Lact (6 × 10^6^ PFU/mouse) reached 160 days since treatment initiation. According to the MRI scans, for these animals, tumors are not visually detected on the 64th day after transplantation. Tumor growth rate was statistically lower in the high-dose virus group compared to the control (Figure 7D).

During histological examination, the tumor nodes of glioblastoma GL261, transplanted orthotopically, in the control group were represented by polymorphic atypical cells of glial origin. The nodes had a round shape with relatively smooth contours, without signs of necrotic destruction. Mild foci of hemorrhage occupying 2.5 ± 0.3% of the total tumor tissue were detected in the control group. The mitotic activity of the tumor is pronounced: the numerical density of mitoses in the tumor tissue was 6.9 ± 0.7 per field of view (Table 5).

**Figure 8:**
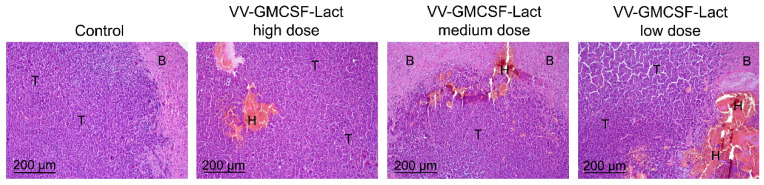
Structural changes in GL261 glioblastoma tissue transplanted orthotopically, without treatment and after intratumoral administration of VV-GMCSF-Lact at high, medium, and low doses. Hematoxylin and eosin staining. Original magnification ×100. T—unchanged tumor tissue, B—brain tissue, H—hemorrhages.

Intratumoural injection of VV-GMCSF-Lact at high (6 × 10^6^ PFU/mouse), medium (5 × 10^6^ PFU/mouse) and low (4 × 10^6^ PFU/mouse) doses did not reveal foci of necrosis in the tumor tissue. However, foci of hemorrhages were detected in the central zone of the tumor node, and the hemorrhages were more massive when the virus was administered at a low dose than at a high or medium dose (Figure 8). However, during the morphometric analysis of tumor nodes, no statistically significant differences between the groups were found: the volume densities of hemorrhages were 10.9 ± 1.8, 6.7 ± 2.2, and 11.8 ± 3.3% of the total tumor tissue upon administration of VV-GMCSF-Lact at high, medium, and low doses, respectively (Table 5).

When assessing tumor mitotic activity, it was revealed that intratumoral administration of VV-GMCSF-Lact led to decreases in the numerical density of mitoses in the tumor tissue in all experimental groups, however, there was an inverse dependence on the administered virus dose. When high, medium, and low doses of virus were administered, the numbers of mitoses were 1.6-, 2.1-, and 5.3-fold lower than in the control group, respectively. Moreover, low-dose virus administration showed a statistically significant difference (3.3-fold) from the group that was administered high-dose virus.

Thus, intratumoral administration of VV-GMCSF-Lact was shown to cause an increase in the hemorrhages in GL261 orthotopic glioblastoma tissue regardless of the dose administered, as well as a significant decrease in mitotic activity, demonstrating an inverse relationship in this case: administration of a low dose of virus caused a more significant decrease in the number of mitoses in the tumor.

## 4. Discussion

Virotherapy is one of the promising immunotherapeutic approaches in treatment of different types of malignancies, including gliomas [18]. Several excellent and up-to-date reviews have provided detailed reviews of current immunotherapeutic strategies and discussed the challenges and potential mechanisms of the molecular basis of immunotherapy resistance in GBM and strategies that can overcome immunotherapy resistance in GBM [29,30,31], which will likely require combination therapies such as virotherapy [32,33,34].

Nowadays, dozens of various oncolytic viruses are in different phases of clinical trials, demonstrating significant outcomes in antitumor efficacy (https://clinicaltrials.gov/, accessed on 30 July 2025). Delytact (G47∆, Teserpaturev) is the first and yet the only approved oncolytic agent for glioma treatment. This drug is based on the herpes simplex virus type 1 strain, in which the *α47* and both copies of *γ34.5* genes have been deleted to reduce neurotoxicity for healthy cells. Delytact also has an insertion of the *E. coli lacZ* gene to disrupt the infectious cellular protein 6 (*ICP6*) gene, which is necessary for viral DNA replication in non-dividing cells. G47∆ demonstrated a survival benefit and safety profile in patients with progressive glioblastoma which led to its approval in Japan in 2021 [20]. Thus, the development of antitumor drugs based on recombinant viruses against malignant neoplasms of the brain is a promising and actively developing direction.

VV-GMCSF-Lact is a prospective double recombinant vaccinia virus designed on the base of the L-IVP strain with deletions of viral thymidine kinase (*tk*) and growth factor (*vgf*) genes and insertions of human GMSCF and peptide lactaptin genes into the regions of these deletions, respectively. These genetic modifications provide selective replication of the virus only in oncotransformed cells and induction of local immune responses. Lactaptin is a fragment of human milk protein kappa-casein with apoptotic properties against various cancer cell cultures [25]. The use of vaccinia viruses (VACVs) as antitumor agents has been previously confirmed in numerous studies of oncolytic drugs [35]. VACVs are characterized by special properties which make them prospective and suitable candidates for oncolytic virotherapy: (a) cytoplasm replication avoiding integration into the host genome; (b) large genome of the virus permitting various genetic modifications; (c) natural tropism and absence of specific entry receptor expanding the panel of targeted tumors; (d) rapid lytic replication cycle leading to effective spread within a tumor [36]. One prominent example of an oncolytic VACV is Pexa-Vec (JX-594, pexastimogene devacirepvec), which, like VV-GMCSF-Lact, has disruption of the *tk* gene and insertion of the *GMSCF* gene and additionally has an insertion of the *lacZ* gene. JX-594 has shown its safety and antitumor efficacy against multiple types of malignancies, including melanoma and hepatocellular and colon carcinoma [37,38]. At the same time, VV-GMCSF-Lact has also demonstrated an encouraging antitumor response against a wide range of tumor cells of varying histogenesis: breast cancer, lung cancer, epidermoid carcinoma, and glioblastoma [23,27]. The virus successfully completed a Phase I clinical trial in patients with recurrent/refractory metastatic breast cancer, and data obtained indicate that VV-GMCSF-Lact is safe and well-tolerated at the dose of 10^8^ PFU (NCT05376527). Earlier it was shown that VV-GMCSF-Lact is able to suppress the growth of glioma cells both in in vitro (immortalized and patient-derived cultures) and in vivo (cell-line-derived and patient-derived xenografts) models [23]. However, it is important to evaluate the effect of the oncolytic virus on immunocompetent animals, since innate and acquired immunity can modulate the action of the oncolytic virus, which in turn indirectly activates the antitumor immune response [35].

The C6 glioma is one of the most common immunocompetent rat glioma models and can be an appropriate imitation of human glioma pathophysiological changes [11]. In the current research we investigated antitumor action of the double recombinant vaccinia virus VV-GMCSF-Lact against rat C6 glioma and compared efficacy of two methods of drug administration (intratumoral or intravenous).

Analyzing two ways of VV-GMCSF-Lact administration, a significant increase in primary tumor volume was observed in the group of intratumoral virus administration compared to the intravenous administration group. Despite the difference in tumor volumes, no differences were observed in the survival of animals between experimental and control groups. It is worth noting that intratumoral VV-GMCSF-Lact administration totally blocked the formation of additional tumor sites compared with the control group and group of intravenous administration. Distant foci of invasive glioma growth, or secondary tumors, located in the region of the 4th ventricle, were observed in the control group and group of intravenous administration, possibly arising from the contribution of the cerebrospinal fluid system, which promotes tumor cell migration and tumor growth [39] (Figure 3B). Also, tumors from the control group and group of intravenous administration were characterized by high levels of cell proliferation markers Ki-67 and fibrillary acidic protein (GFAP). The proliferative activity of tumor cells is a prominent parameter to assess malignancy and predict the results of suggested treatment; in this context, the important and sensitive marker Ki-67 is used, reflecting the rate of cell proliferation [40]. GFAP can be a marker of proliferative activity, particularly in the context of glial cells, but, moreover, it is also a part of the intermediate filament of astrocytes and the main indicator of reactive astrocytes [41]. Reactive astrocytes are known for their prominent role in malignant tumors of the CNS because of their involvement in many protumor functions. Zhang, Chuan-Bao et al. demonstrated in their work the negative correlation between the amount of reactive astrocytes and overall survival in patients with diffuse astrocytoma and glioblastoma: the higher the quantity of reactive astrocytes, the shorter overall survival [42].

It is noteworthy that animals from the intratumoral injection group were characterized by no secondary tumor sites, which can be attributed to a more effective impact of the virus on the tumor mass with this type of drug administration. Moreover, in brains of only the intratumorally injected animals, extensive areas of necrotic decay and massive microcellular infiltration were observed. Therefore, the significant tumor volume increase in the group of intratumoral administration can be explained by MRI- and histology-confirmed edema caused by massive tumor destruction and mixed-cell infiltration. Remarkably, immunohistochemical analysis verified the extremely low levels of Ki-67 and GFAP markers in tumors of this group, which may indicate a decrease in the tumor cell proliferation rate and the number of reactive astrocytes by intratumoral injection of an oncolytic virus. Thus, the absence of secondary tumors, extensive tumor tissue decay and immune cell infiltration, the reduction of cell proliferation rate, and amount of reactive astrocytes, indicated by low levels of GFAP, allow us to conclude that the effectiveness of the oncolytic virus is increased with intratumoral administration of the drug compared to intravenous administration to animals.

Unfortunately, the chosen dose of intratumoral virus injection led to the early death of 55.5% of the whole animal group—from the second day after the start of the treatment. This phenomenon is possibly caused by the appearance of side toxic effects, which include acute tumor necrosis, local brain edema, and inflammation. The occurrence of toxic effects can be explained by various reasons. First of all, in the study of recombinant vaccinia virus JX-594 the destruction of the tumor mass with subsequent GMCSF-dependent necrosis and edema formation was shown. Possibly, as in our vaccinia virus study, the enhanced inflammatory response may have been a result of viral *GMCSF* expression and not the direct oncolytic virus replication, however, this requires future research beyond the scope of this paper [43]. Further, necrotic cells release factors like high mobility group box 1 protein (HMGB1), which stimulate neighboring cells to express proinflammatory cytokines, leading to inflammation. The inflammatory response attracts leukocytes and phagocytes, and this creates collateral damage to surrounding tissues [44]. The larger the tumor, the more serious the necrotic damage. Injection of JX-594 into the normal rodent brain is associated with diffuse inflammation [43]. According to our results, when VV-GMCSF-Lact was administered into the brain of tumor-free rats, we observed signs of inflammation on the MRI and poor cell infiltration, confirmed by histological analysis. In addition to the above reasons, early mortality can be also attributed to factors related to rats (biological variety, stress, or exhausting work)—32%, mortality due to anesthesia—23%, mortality in controlled laboratory conditions (cages, social organization, transport, handling, and water)—26%; the lowest mortality was attributed to environmental factors—19% [45]. These reasons may also be a cause of bias in the experimental results. All tumor-free rats that were injected with the virus intratumorally or intravenously showed no signs of adverse side effects. The results obtained revealed an urgent need for experiments to select the dose of the virus, which we actually performed on a syngeneic murine glioma model, GL261.

Despite this, in the group with intratumoral administration of the drug, there was a rat whose lifespan exceeded 60 days from the start of treatment. This rat received a lower dose of the virus at the first administration due to the reflux of a part of the drug. Perhaps, starting treatment with a lower dose of the drug with its further escalation will make it possible to reduce side toxic effects. Similar results were obtained for herpes simplex virus G47Δ versus human glioblastoma stem cell xenografts [46]. The results obtained revealed an urgent need for experiments to select the dose of VV-GMCSF-Lact. This study was conducted on an immunocompetent mouse model of glioma GL261.

The GL261 murine glioma is also one of the most commonly used immunocompetent models that reflects many pathological characteristics of human gliomas. Thus, GL261 is hallmarked by pleomorphic cells with atypical nuclei, a high degree of neoangiogenesis, increased mitosis rate, hypoxia, and extremely reduced lymphocyte infiltration, representing the low immune status in human gliomas [47,48]. When studying the optimal VV-GMCSF-Lact dose regimen during intratumoral administration in C57Bl/6 mice with orthotopically transplanted murine glioma GL261 it was shown that tumor volumes did not differ between control and experimental groups during the treatment process. However, the group of the high virus dose (6 × 10^6^ PFU/mouse) is characterized by the highest animal survival: two mice survived more than 160 days compared with the control group and groups that received the low and medium doses of the viral drug and are still alive at the time of writing. Moreover, tumor growth rate was statistically lower in the high-dose virus group (6 × 10^6^ PFU/mouse) compared to the control.

Histological analysis of GL261 tumors from virus-treated mice demonstrates the decreasing of mitotic tumor cells compared with the control group. Early genes of VACV replication, particularly early viral kinase B1, can arrest the cell cycle by hyperphosphorylating the p53 protein and its degradation, which in turn activate the p21 cell cycle inhibitor, arresting the cell cycle in the S/G2 stage [49]. The described cell cycle shift provides more effective viral replication and inhibition of the tumor cell growth.

It is worth noting that no adverse toxic events were detected in the experiment of optimizing the therapeutic dose. We propose that VV-GMCSF-Lact at a dose of 6 × 10^6^ PFU can be considered optimal and safe for intratumoral administration for the treatment of glioma, despite the fact that, at a dose of 4 × 10^6^ PFU, there was a more significant decrease in mitoses. This occurs because, at high doses, there is a tendency to increased life expectancy and there are smaller areas of hemorrhage as opposed to those seen with a low dose, which may have undesirable consequences.

The problem of treating malignant neoplasms of the brain lies not only in finding a suitable treatment but also in the problem of the outflow of exudate from the brain area. Since perivascular and pericellular edemas were observed in the brains of rats and mice in different study groups, it can be speculated that the accumulation of exudate may have been a consequence of direct tumor formation rather than ongoing treatment. Neoplasm blood vessels show abnormal structure, carrying increased blood volume, and a disrupted blood–brain barrier allows plasma exudate to leak into the surrounding brain tissue, causing undesirable inflammation and major edema [50]. Given the specific properties of malignant brain neoplasms, it is necessary to select a treatment modality taking into account not only the patient’s susceptibility to the therapy but also the possibility of minimizing toxic side effects.

## 5. Conclusions

In this work, we evaluated the effectiveness of the oncolytic virus VV-GMCSF-Lact on a model of immunocompetent rats with orthotopic transplanted C6 glioma with intravenous and intratumoral administration of the drug. Despite the increase in rat C6 tumor volumes after intratumoral VV-GMCSF-Lact injections due to the emergence of mixed-cell infiltration and pericellular and perivascular edemas, a significant decrease in the level of the proliferation marker Ki-67 was observed compared with the control group and group of intravenous administration. The notable toxic side effects with intratumoral injection of the drug required a revision of the treatment scheme, and an additional experiment on a model of immunocompetent mice glioma GL261 was carried out to select VV-GMCSF-Lact doses to show no toxic adverse events. Meanwhile, the life expectancy of two mice, who received 6 × 10^6^ PFU of VV-GMCSF-Lact, reached 160 days since the treatment initiation. According to the study of another recombinant vaccinia virus, JX-594, where possibly the expression of *GMCSF* led to the formation of extensive inflammation, it is necessary to test the impact of this cytokine in future studies to identify its direct involvement in the antitumor effect of the oncolytic virus. From our point of view, the most optimal option in the primary stage of glioblastoma treatment is surgical resection of the tumor, and the use of a virus to treat the resection field will destroy the remaining tumor cells and minimize the toxic effect. A similar modality has been used in several studies of different oncolytic drugs against glioma, demonstrating antitumor efficacy and increased overall patient survival, with minimal adverse events [51] (NCT03072134, NCT01301430). Oncolytic virotherapy in combination with chemotherapy, radiotherapy, and anti-inflammatory drugs also allows for a reduction in the drug dose. The challenges we have encountered in this work confirm the difficulty of choosing a treatment method for malignant brain tumors and also allow us to outline a range of problems that will help us get closer to the desired result. Nevertheless, VV-GMCSF-Lact is a promising agent for glioma therapy because it demonstrates potent and rapid antitumor efficacy with intratumoral delivery confirmed by histology and immunohistochemical analysis. Thereby, the data obtained in this and our previous studies support that the double recombinant vaccinia virus VV-GMCSF-Lact is a candidate for further preclinical and clinical trials against malignant brain tumors.

## Figures and Tables

**Table 1:** The scheme of experiment of VV-GMCSF-Lact antitumor efficacy against C6 rat glioma in vivo.

Group	Administration Way	Virus Dose	Administration Scheme
A (i.t.)	Intratumoral (i.t.)	1.65 × 10^7^ PFU	3 injections every 7 days
B (i.v.)	Intravenous (i.v.)	3 × 10^8^ PFU	3 injections every 7 days
C (Control)	-	-	-
D (Intact control)	Intracerebral/intravenous	1.65 × 10^7^ PFU or 3 × 10^8^ PFU, respectively, in each administration route	1 injection

**Table 2:** The scheme of experiment of VV-GMCSF-Lact antitumor efficacy against GL261 murine glioma in vivo.

Group	Virus Dose	Administration Scheme
E (High dose)	6 × 10^6^ PFU	3 intratumoral injections every 7 days
F (Medium dose)	5 × 10^6^ PFU	3 intratumoral injections every 7 days
G (Low dose)	4 × 10^6^ PFU	3 intratumoral injections every 7 days
H (Control)	Saline solution	3 intratumoral injections every 7 days

**Table 3:** Rat C6 glioma additional tumor site distribution without treatment and during the VV-GMCSF-Lact therapy.

Group	20 Days	26 Days
i.t. (A)	0/4 *	0/4
i.v. (B)	7/9	3/4
Control (C)	2/2	-

i.t. (A)—intratumoral VV-GMCSF-Lact administration; i.v. (B)—intravenous VV-GMCSF-Lact administration; control (C)—rat C6 glioma without treatment. Days are indicated since the tumor cell transplantation. Differences from the i.v. administration (*) were considered statistically significant at *p*-value of 0.05 (Pearson chi-square test).

**Table 4:** Morphological changes in rat C6 glioma without treatment and after VV-GMCSF-Lact administration.

Morphological Parameter, Vv, %	Control	VV-GMCSF-Lact i.v.	VV-GMCSF-Lact i.t.
Unaltered tumor tissue	88 ± 2.8	68.6 ± 4.1 *	4.7 ± 1.2 *#
Cell infiltration	9 ± 2.9	26.7 ± 3.9 *	51 ± 15.6 *#
Necrosis	3 ± 1.5	4.7 ± 1.9	44.4 ± 14.4 *#

Control—rat C6 glioma without treatment; i.v.—intravenous VV-GMCSF-Lact administration; i.t.—intratumoral VV-GMCSF-Lact administration. Vv %—the volume density representing the volume fraction of tumor tissue occupied by the studied compartment. Differences from the control (*) and i.v. administration (#) were considered statistically significant at *p*-value of <  0.05.

**Table 5:** Mitotic activity of GL261 glioblastoma transplanted orthotopically, without treatment and after intratumoral injection of VV-GMCSF-Lact at high, medium, and low doses.

Morphological Parameter	Control	VV-GMCSF-Lact		
		High Dose	Medium Dose	Low Dose
Mitoses, Nv	6.9 ± 0.7	4.3 ± 0.6 *	3.3 ± 0.7 *	1.3 ± 0.5 **#
Hemorrhages, Vv, %	2.5 ± 0.3	10.9 ± 1.8	6.7 ± 2.2	11.8 ± 3.3

Nv—numerical density of mitoses per unit of field of view of tumor tissue. Vv, %—the volume density representing the volume fraction of tumor tissue occupied by the hemorrhages. Statistically significant differences from the control at * *p* ≤ 0.05, ** *p* ≤ 0.01. Statistically significant differences from high dose at # *p* ≤ 0.05.

## Data Availability

The original contributions presented in this study are included in the article/Supplementary Material. Further inquiries can be directed to the corresponding author(s).

## Supplementary Materials

The following supporting information can be downloaded at: https://www.mdpi.com/article/10.3390/cells14201619/s1, Figure S1. DWI MRI comparison of animals with lifespan less than 30 days and rat that received VV-GMCSF-Lact intratumorally and whose survival was more than 30 days. Figure S2. Structural changes in C6 glioma, transplanted orthotopically into the brain of rats, after administration of VV-GMCSF-Lact intravenously (i.v.) or intratumorally (i.t.). Figure S3. Structural changes in the brain of non-tumor-bearing healthy rats after administration of VV-GMCSF-Lact intracerebrally.

## Abbreviations

The following abbreviations are used in this manuscript:

GBM	Glioblastoma
IGF	Insulin-like growth factor
FGF	Fibroblast growth factor
TGF	Transforming growth factor
PTEN	Phosphatase and tensin homolog deleted on chromosome 10
PI3K	Phosphoinositide 3-kinase
TIME	Tumor microenvironment
TNF	Tumor necrosis factor
IFN	Interferon
MHC	Major histocompatibility complex
GMCSF	Granulocyte–macrophage colony-stimulating factor
PFU	Plaque-forming unit
MRI	Magnetic resonance imaging
DWI	Diffusion-weighted imaging
tk	Thymidine kinase
vgf	Virus growth factor
TGI	Tumor growth inhibition
GFAP	Glial fibrillary acidic protein
Ki-67	Antigen Kiel 67
ICP6	Infectious cellular protein 6
