# Supplementary material for: Efficacy of Oncolytic Virus VV-GMCSF-Lact Against Immunocompetent Glioma
Source: Cells. 2025 Oct 17;14(20):1619. doi: 10.3390/cells14201619 (PMC12562511; doi:10.3390/cells14201619)
Supplement: Supplementary file 1 [file cells-14-01619-s001.zip › cells-3841077-supplementary.pdf]

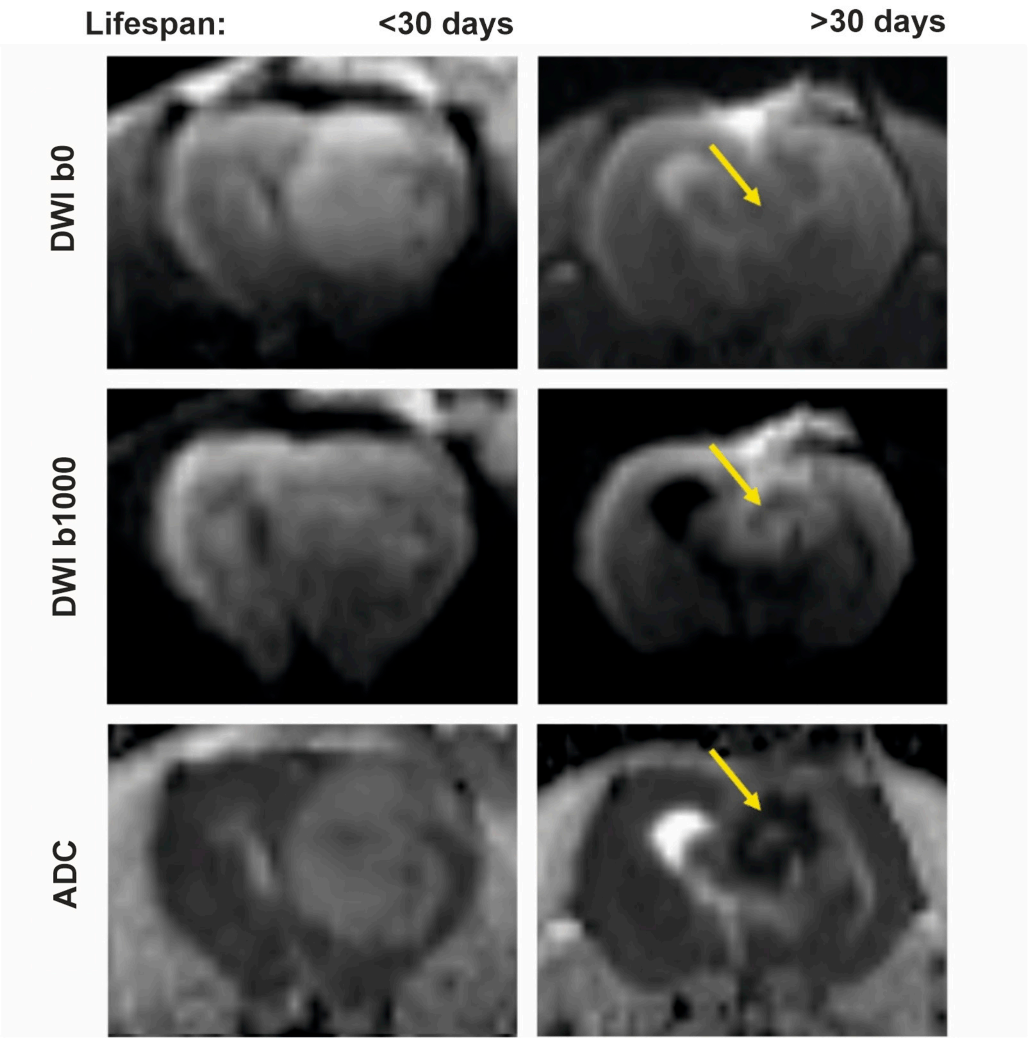

Figure S1. DWI MRI comparison of animals with lifespan less than 30 days and rat that received VV-GMCSF-Lact intratumorally and whose survival was more than 30 days. In the first animal, a T2-hyperintense lesion is visualized, diffusely accumulating MR contrast agent. In the second animal, in the area of tumor cell transplantation, a structure with a thick wall and restriction diffusion of water molecules and a site of relative increased intensivity in the center is visualized, with accumulation of contrast agent along the wall, without signs of hemorrhage in the structure (DWI b0 partially has T2* weighting) - yellow arrow.

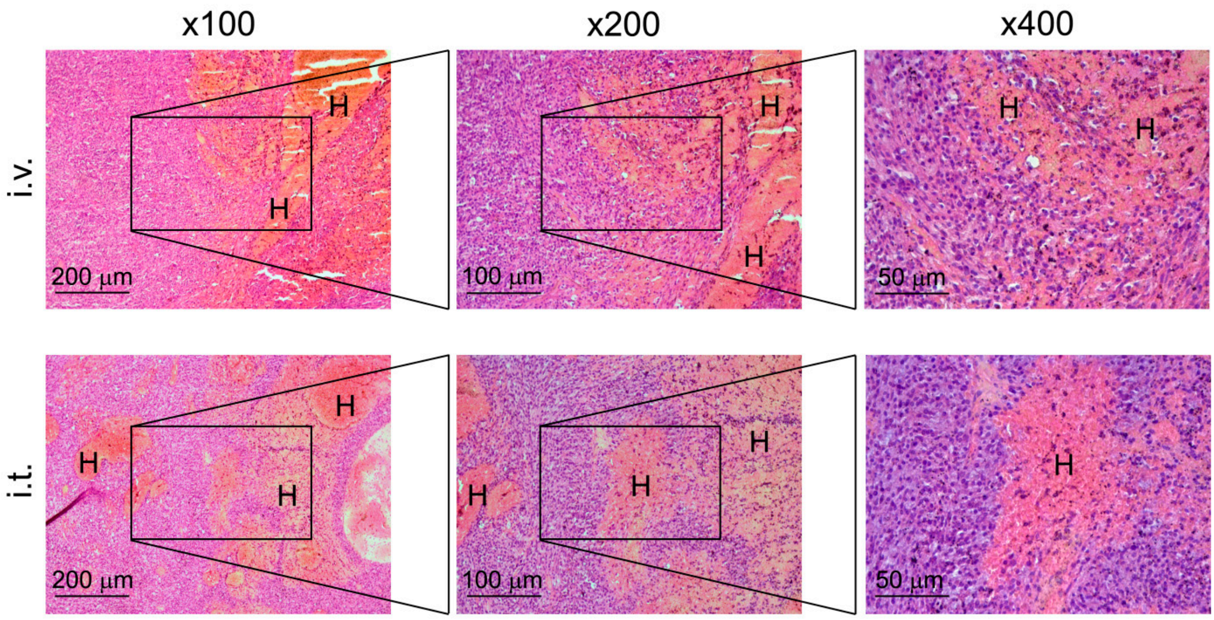

Figure S2. Structural changes in C6 glioma, transplanted orthotopically into the brain of rats, after administration of VV-GMCSF-Lact intravenously (i.v.) or intratumorally (i.t.). Hematoxylin and eosin staining. Original magnification ×100 (left panel), ×200 (middle panel), and ×400 (right panel). The black boxes show areas that were examined further at a higher magnification. H – hemorrhages.

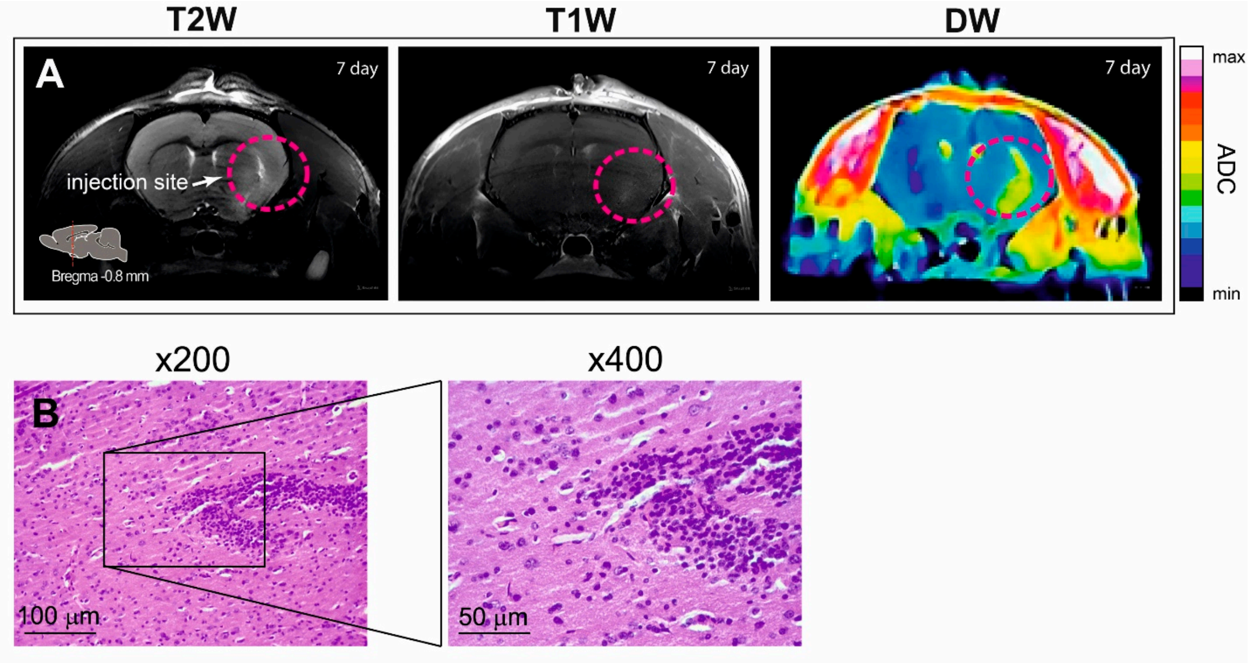

Figure S3. Structural changes in the brain of non-tumor-bearing healthy rats after administration of VV-GMCSF-Lact intracerebrally. (A) MRI of non-tumor-bearing healthy animals on the 7th day after the intracerebral injection of VV-GMCSF-Lact. Based on the T1, T2, DW-weighted MRI data obtained, only a needle track after the virus injection and mild edema of the hemisphere, where the injection was performed, were observed. (B) Hematoxylin and eosin staining. Original magnification ×200 (left panel) and ×400 (right panel). The black boxes show areas that were examined further at a higher magnification.
